# Patient Reported Outcome (PRO) assessment in epilepsy: a review of epilepsy-specific PROs according to the Food and Drug Administration (FDA) regulatory requirements

**DOI:** 10.1186/1477-7525-11-38

**Published:** 2013-03-11

**Authors:** Annabel Nixon, Cicely Kerr, Katie Breheny, Diane Wild

**Affiliations:** 1 Oxford Outcomes, an ICON Plc. Company, Seacourt Tower, West Way, Oxford, OX2 0JJ, UK

**Keywords:** Patient reported outcomes (PRO), Epilepsy, Review, FDA regulatory requirements

## Abstract

Despite collection of patient reported outcome (PRO) data in clinical trials of antiepileptic drugs (AEDs), PRO results are not being routinely reported on European Medicines Agency (EMA) and Food and Drug Administration (FDA) product labels. This review aimed to evaluate epilepsy-specific PRO instruments against FDA regulatory standards for supporting label claims. Structured literature searches were conducted in Embase and Medline databases to identify epilepsy-specific PRO instruments. Only instruments that could potentially be impacted by pharmacological treatment, were completed by adults and had evidence of some validation work were selected for review. A total of 26 PROs were reviewed based on criteria developed from the FDA regulatory standards. The ability to meet these criteria was classified as either full, partial or no evidence, whereby partial reflected some evidence but not enough to comprehensively address the FDA regulatory standards. Most instruments provided partial evidence of content validity. Input from clinicians and literature was common although few involved patients in both item generation and cognitive debriefing. Construct validity was predominantly compromised by no evidence of a-priori hypotheses of expected relationships. Evidence for test-retest reliability and internal consistency was available for most PROs although few included complete results regarding all subscales and some failed to reach recommended thresholds. The ability to detect change and interpretation of change were not investigated in most instruments and no PROs had published evidence of a conceptual framework. The study concludes that none of the 26 have the full evidence required by the FDA to support a label claim, and all require further research to support their use as an endpoint. The Subjective Handicap of Epilepsy (SHE) and the Neurological Disorders Depression Inventory for Epilepsy (NDDI-E) have the fewest gaps that would need to be addressed through additional research prior to any FDA regulatory submission, although the NDDI-E was designed as a screening tool and is therefore unlikely to be suitable as an instrument for capturing change in a clinical trial and the SHE lacks the conceptual focus on signs and symptoms favoured by the FDA.

## Introduction

Epilepsy is defined by the recurrence of spontaneous/unprovoked seizures and covers a range of clinical situations in terms of age of onset, type of seizures, aetiological background, resulting handicap, prognosis, and response to treatment [1]. Epilepsies are a diverse group of disorders with a complex classification, broadly categorised into localization-related, generalized, undetermined, and special syndromes [2]. Antiepileptic drugs (AEDs) are the main treatment option; approximately 60% of newly diagnosed patients are seizure-free with AED monotherapy and a further 10-20% with polytherapy [1,3]. Surgery offers alternative treatment options for patients with medically intractable epilepsy [4].

The assessment of efficacy in clinical studies evaluating AEDs is generally focused on seizure frequency/occurrence in line with regulatory guidelines [1]. However there is growing recognition of the value of capturing wider impacts of treatments reported by patients in the form of patient reported outcomes (PROs) [5]. A PRO is defined as any report of the status of a patient’s health condition that comes directly from the patient without interpretation of the patient’s response by a clinician or anyone else [5]. PROs take the form of carefully designed questionnaires which can be used to capture and quantify the patient experience of treatment and treatment impacts. Given that epilepsy is a complex disorder, which affects patients’ psychological health, independence, emotional adjustment and employment [6] there is a strong case for evaluating the impact of AEDs on broader aspects of patient’s lives, and previous studies have identified potential areas of patient’s lives that may be enhanced if epilepsy symptoms were improved [7]. Therefore it is unsurprising that PROs have been widely incorporated into clinical trials evaluating AEDs [8-10].

In order to guide the selection of PROs for epilepsy clinical studies, there have been numerous reviews evaluating PROs, each review with a unique focus: evaluating PROs designed to measure change in seizure severity [11], overview of neuropsychological and behavioural measures used AED clinical trials [8], guiding health-related quality of life (HRQL) assessment in epilepsy [12], systematically reviewing measures designed to assess the subjective impact of epilepsy and treatment in children and adolescents [13], guiding the selection and use of quality of life (QOL) instruments in epilepsy [9], appraising the conceptual underpinnings of paediatric QOL instruments [14], exploring and reviewing PROs used to assess people with epilepsy [10], and describing QOL instruments for children and adolescents with neurodisabilities [15]. Similar reviews were also conducted prior to 2000 [16,17].

Despite almost routine collection of PRO data in clinical trials of AEDs and the wide choice of PRO instruments, PRO results are not being routinely reported on European Medicines Agency (EMA) and Food and Drug Administration (FDA) product labels. A review of the current state of PRO label claims granted for new molecular entities approved 2006–2010 [18] found two approved for the treatment of epilepsy had PRO label claims: Vimpat® indicated as adjunctive therapy for patients ≥17 years to treat partial onset seizures and Sabril® indicated for refractory complex partial seizures in adults. For both products the PRO was a measure of seizure frequency. There are two likely explanations for the lack of PRO label claims for epilepsy treatments. The first relates to the possibility that there may be a lag in the effect of AED treatment on PRO outcomes after demonstration of positive treatment effect on clinical efficacy outcomes e.g. seizure frequency/occurrence. Qualitative studies show that unpredictability of seizures is a key factor in the impact of epilepsy for patients [19-21]. Patients’ perception of the unpredictability of their seizures may not change until sometime after a reduction in seizure frequency, or even seizure freedom is achieved; 3–4 months, a common timescale for AED efficacy trials, may be too short to expect much change in PRO endpoints [11]. The second explanation, and the focus of the current review, is the possibility that existing PRO instruments do not meet regulatory requirements.

There is limited guidance from regulatory authorities on incorporating PROs into evaluations of AEDs, the recent EMA guideline on clinical investigation of medicinal products in the treatment of epileptic disorders [1] suggests that a secondary efficacy variable could be ‘scales measuring social and working capacity, if validated’. Other than this the authors could find no further guidance from either the EMA or FDA specific to the use of PROs in epilepsy clinical studies. For a PRO to be incorporated into a FDA or EMA label claim, it is necessary for it to be developed to standards required by the regulatory authorities [5,22]. Most of the epilepsy-specific PRO reviews conducted to date were published prior to the current regulatory guidance, and none have evaluated the extent to which PROs have been developed to the standard required by regulatory authorities. In order to guide those developing clinical studies to evaluate AEDs in selecting a PRO instrument for the purpose of obtaining a PRO label claim, this review sought to evaluate existing epilepsy-specific PRO instruments against FDA regulatory standards and identify any gaps in the development process that would need to be addressed prior to regulatory review.

## Methods

### Literature search and PRO identification

Structured Embase and Medline searches were conducted in February 2011 to identify epilepsy studies reporting the use or development of PRO instruments in epilepsy samples. Searches were constructed by combining epilepsy and seizure subject heading search terms with a number of PRO measurement terms. Searches were limited to ‘humans’, ‘English language’ and ‘year 2000 –current’. Full search terms are shown in the Additional file 1.

Abstracts of publications identified by these searches were screened to identify PRO instruments used. Full text versions of publications were screened to identify specific instruments where abstracts referred to PRO measurement without naming a specific instrument, or where abstracts specified endpoints that may have been captured by PRO measurement. Further electronic searches were conducted for each instrument identified to retrieve a copy of the instrument and establish its relevance. Given the wide variety of PRO instruments used with epilepsy patients, the following instrument inclusion criteria were used to focus the review on broadly comparable instruments that might be used to evaluate pharmacological treatment:

•Epilepsy-specific scale. Based on the FDA’s preference for more proximal endpoints that are specifically relevant to the target population, generic PROs which make no reference to epilepsy were excluded.

•Adult patient completed. Most epilepsy-specific PROs have been developed for adult patient completion. Scales developed to assess epilepsy impacts for children are commonly designed for proxy completion (e.g. by parents or clinical assessors) in part or whole and are not directly comparable to PROs designed for adult patient completion. PROs designed for completion by adolescent as well as adult patients were included.

•Target a concept that could potentially be impacted by pharmacological treatment. Non-interventional studies or those evaluating non-pharmacological interventions may also use PROs to evaluate concepts that would not be impacted by pharmacological treatment. However, PROs evaluating these concepts would not need to meet FDA requirements for supporting a label claim.

Further literature searches were then conducted in Embase and Medline to identify published validation work on each PRO instrument identified. Full text versions of all published reviews of PRO instruments identified by the initial search and instrument focused literature searches were retrieved and checked for any additional instruments or validation work. Any instrument that was found to comprise single items that did not form a scale and/or for which no documentary evidence of validation could be located was excluded from further review.

### Developing the review criteria

Detailed review criteria were developed from requirements laid out in the FDA guidance for PROs supporting label claims [5]. These criteria are detailed in Table 1. Development work and evidence for psychometric properties of each PRO instrument were extracted into structured individual instrument review tables before being systematically reviewed against the review criteria (Table 1).

**Table 1:** Review criteria based on FDA requirements for PROs to support label claims

**Instrument property**	**Description from FDA guidance**[5]	**Review criteria**	
		**Notation**	**Detail**
Conceptual Framework	An explicit description or diagram of the relationships between items in a PRO instrument and the concepts measured, developed from empiric evidence to support item grouping and scores.	✓	Published conceptual framework.
		✘	No published conceptual framework.
Validity: Content – Patient Input	Evidence that the instrument measures the concept of interest including evidence from qualitative studies that the items and domains of an instrument are appropriate and comprehensive relative to its intended measurement concept, population and use. Item generation should include input from the target population.	✓	Patient involvement in concept elicitation/item generation AND conduct of cognitive debrief with patients.
		Partial	Some patient involvement in concept elicitation/item generation or cognitive debrief but not both.
		✘	No patient involvement in instrument development and evaluation of content.
Validity: Content – Literature & Clinician Input	See Validity: Content (above). In addition to focus groups and individual interviews with patients and family members, PRO instrument items can be generated from literature reviews, interviews with clinicians and other sources.	✓	Use of literature to guide instrument/item development OR involvement of clinical experts to guide instrument/item development or evaluate content validity (not necessary to have both).
		✘	No use of literature to guide instrument/item development AND no involvement of clinical experts to guide instrument/item development or evaluate content validity.
Validity: Construct	Evidence that relationships among items, domains, and concepts conform to *a priori* hypotheses concerning logical relationships that should exist with other measures (discriminant and convergent validity) or characteristics of patients and patient groups (known groups validity).	✓	Clear hypotheses for relationships with measures (PRO or clinical) of related concepts tested with hypothesised relationships found (convergent validity). Can be supported by evidence that measures of concepts that should not be related show hypothesised lack of correlation (discriminant validity). Can also be supported by known groups validity (hypothesised, tested and found) but known groups validity alone is not sufficient evidence of construct validity.
		Partial	Construct validity tested without clear hypotheses OR mixed results in terms of the extent to which observed relationships match those hypothesised OR limited number of tests undertaken i.e. if instrument is correlated against one other PRO and that’s the extent of the testing, then this is only partial evidence of construct validity.
		✘	Construct validity tested but observed relationships do not match those hypothesised.
		–	Construct validity (convergent, discriminant and known groups validity) not tested.
Reliability: Test-retest	Stability of scores over time when no change is expected in the concept of interest.	✓	Correlations ≥0.7 for all scores (including domain scores)
		Partial	Correlations for some scores <0.7 OR good test-retest reliability found for total score but domain scores not evaluated.
		✘	Correlations <0.7 for all scores evaluated.
		–	Not tested for any scores.
Reliability: Internal consistency	The extent to which items comprising a scale measure the same concept, intercorrelation of items that contribute to a score.	✓	Cronbach’s Alpha ≥0.8 for all scores (including domain scores).
		Partial	Cronbach’s Alpha for some scores <0.8 OR good internal consistency found for total score but domain scores not evaluated.
		✘	Cronbach’s Alpha <0.8 for all scores evaluated.
		–	Not tested for any scores.
Ability to detect change	Evidence that a PRO instrument can identify within person changes over time in individuals or groups (similar to those in clinical trials) who are known to have changed with respect to the measurement concept.	✓	Specific aim of analysis was to test within-group responsiveness to change (e.g. set criteria for change e.g. effect sizes), tested and met criteria for all scores (including domain scores). Of key importance is clear evidence/reason to believe that change has occurred in a group (e.g. clinical outcome, anchor-based approach) and that the PRO instrument scores detect this change.
		Partial	Within-group sensitivity to change criteria met for some but not all scores OR criteria met for a total score but responsiveness of domain scores not tested.
		✘	Within group sensitivity to change tested but criteria not met.
		–	Not tested for any scores. This includes claims of instruments sensitivity to change based on between group change (e.g. difference in change between different arms of clinical trial) and observed change in a group without clear evidence/reason to believe that change has occurred in the group or without the clear aim of evaluating sensitivity to change (e.g. observed change from baseline within one arm of a clinical trial when not evaluated in relation to observed clinical change).
Interpretation of change	The MID is the smallest change in score that can be regarded as important [23]. The FDA guidance uses the term ‘responder definition’ rather than MID to denote the change in individual PRO score that indicates a treatment benefit. Responder definitions are trial/treatment specific and should be derived empirically using anchor-based methods (clinical anchors or patient ratings of change). Statistically derived responder definitions (e.g. distribution-based methods commonly used to establish MID) can be used to support anchor-based approaches but are not appropriate as the sole basis for determining a responder definition.	✓	Published values for interpretation of change for all scores (including domain scores). Methodological details about how values were derived e.g. statistically, using anchor-based methods, provided and discussed in results text.
		Partial	Values for interpretation of change for total score but not domain scores. Methodological details about how this was derived e.g. statistically, using anchor-based methods, provided and discussed in results text.
		–	No published evidence for interpretation of change.

Glossary of Terms:

*Cognitive debrief:* a qualitative research tool used to determine whether concepts and items are understood by patients in the same way that instrument developers intend.

*Concept:* the specific measurement goal (i.e. the thing that is to be measured by the PRO instrument).

*Item:* an individual question, statement or task (and its standardized response options) that is evaluated by the patient to address a particular concept.

*Reliability:* the ability of a PRO instrument to yield consistent, reproducible estimates of true treatment effect.

*Responder definition:* a score change in a measure, experienced by an individual patient over a predetermined time period that has been demonstrated in the target population to have significant treatment benefit.

## Results

### Identification of PROs

Initial Embase and Medline searches identified a total of 1854 publications (after de-duplication) from which 159 PRO instruments were identified by abstract or full-text manuscript review. Following instrument retrieval and initial review, 133 of these were excluded as they were found to be not epilepsy specific (n = 52), not for adult patient completion (n = 42), not to target a concept that could be potentially impacted by pharmacological treatment (n = 20) or instruments comprising single items that did not form a scale and/or for which no documentary evidence of validation could be located (n = 19). Many of these instruments could be excluded on more than one criterion. Numbers shown here depict the first reason for exclusion.

### PRO instrument characteristics

Twenty-six (26) epilepsy-specific PRO instruments were identified and reviewed, Table 2 details the key characteristics for each of these 26 instruments. The identified PROs vary widely in their key characteristics. Conceptual coverage of the PROs include instruments designed to capture epilepsy attack experience including severity (e.g. Attack Symptom Measure, Ictal Consciousness Inventory (ICI), Liverpool Seizure Severity Scale (LSSS), Seizure Severity Questionnaire (SSQ)); issues associated with the treatment and management of epilepsy (e.g. Assessing Side Effects of AED Treatment (SIDAED), Aldenkamp-Baker Neuroassessment Schedule (ABNAS), Liverpool Adverse Events Profile (LAEP), Portland Neurotoxicity Scale (PNS), Epilepsy Self-Efficacy Scale (ESES)); instruments designed to capture the impacts of epilepsy on different aspects of patient’s lives (e.g. Epilepsy Psycho-Social Effects Scale, Impact of Epilepsy Questionnaire, Neurological Disorders Depression Inventory for Epilepsy (NDDI-E), Perceived Stigma Scale, Washington Psychosocial Seizure Inventory (WPSI)) and instruments measuring the impact of epilepsy on patients’ HRQL (e.g. Epilepsy Foundation of America (EFA) Concerns Index, Epilepsy Surgery Inventory 55 (ESI-55), EPI-QOL, Quality of Life in Epilepsy (QOLIE) instruments, Quality of Life in Newly Diagnosed Epilepsy (NEWQOL)).

**Table 2:** Epilepsy-specific PRO instruments

**Abbreviated name**	**Full name**	**Overview**	**Scoring: total score & sub-scales**	**Number of items**	**Recall period**	**Response options**	**Published details of instrument development and psychometric properties**
ABNAS	Aldenkamp-Baker Neuroassessment Schedule	An instrument to measure patient perceived cognitive side effects of AED treatment.	Total score and 6 subscale scores:	24	No recall period	Four point scale ranging from ‘no problem’ to ‘a serious problem’	[24-28]
			1. Fatigue				
			2. Slowing				
			3. Memory				
			4. Concentration				
			5. Language				
			6. Motor coordination				
	Attack Symptom Measure	An instrument to measure ictal symptoms that would typically be associated with panic disorder.	Total score and five sub-scales:	26	During their attacks	Two options indicating the absence or presence of the symptom	[29]
			1. Autonomic arousal symptoms				
			2. Chest and abdomen symptoms				
			3. Mental state symptoms				
			4. General symptoms				
			5. Cognitive symptoms				
BPSE	Bonner Psychsoziale Skalen fur Epilepsie	A self-report questionnaire designed to evaluate the patient’s areas of illness-related psychosocial problems and cognitive-behavioural variables.	20 subscales in six areas:	87	No recall period in example items reviewed	Analogue ratings of between 0 to 6. Scales include Never to Always or Not at all to Extremely and primarily focus on frequency of cognitions or behaviour	[30]
			1. Impairment to daily life				
			a. Physically				
			b. Activity/capability				
			c. Relations and family				
			d. Emotional/mood				
			e. Independence				
			f. Problem-solving/coping				
			2. Environmental experiences				
			a. Positive				
			b .Negative				
			c. Present emotions/moods				
			3. Attitude to illness/seizures				
			a. Helplessess				
			b. External attribution				
			c. Internal attribution				
			4. Relatives’ reaction to seizures				
			a. Calming down/relief				
			b. Punishment				
			c. Distraction				
			5. Activities				
			a. Home/family				
			b. Social				
			c. Cultural				
			6. Influences on seizure occurrence				
			a. Personal/others				
			b. Situational				
EFA concerns index	Epilepsy Foundation of America concerns index	A HRQL measure for patients with epilepsy, covering concerns relating to affective enjoyment, general autonomy, seizure recurrence, family burden and lack of understanding.	Ratings are summed to produce a Concerns Index Score.	20	Past 4 weeks	Five point scales ranging from ‘not at all concerned’ to ‘extremely concerned’ or ‘none of the time’ to ‘all of the time’	[31-33]
EPSES	Epilepsy Psycho-Social Effects Scale	An instrument to determine the social effects of epilepsy and measure psychosocial functioning.	Total weighted score and 14 subscale scores:	42	No recall period	Five point scale ranging from ‘almost always or always’ to ‘never’	[34]
			1. Attitude towards accepting attacks				
			2. Fear of having seizures				
			3. Fear of stigma in employment				
			4. Lack of confidence about the future				
			5. Lack of confidence about travelling				
			6. Adverse reaction on social life				
			7. Adverse reaction on leisure pursuits				
			8. Change in outlook on life/self				
			9. Difficulty communicating with the family				
			10. Problems with taking medication				
			11. Distrust of the medical profession				
			12. Depression or emotional reactions				
			13. Feeling of increased social isolation				
			14. Lethargy/lack of energy				
-	EPI-QOL	A HRQL assessment instrument for adults with epilepsy.	Total score and six subscale scores:	46	Past two weeks and now	Five point scale ranging from ‘very frequently’ to ‘not at all’.	[35]
			1. Physical functioning				
			2. Emotional wellbeing				
			3. Cognitive functioning				
			4. Social functioning				
			5. Seizure worry				
			6. Medication effects				
ESES	Epilepsy Self-Efficacy Scale	An instrument to measure self-efficacy in regards to epilepsy management.	Total score.	33	No recall period	Ten point scale ranging from ‘I cannot do at all’ to ‘Sure I can do’	[36,37]
ESI-55	Epilepsy Surgery Inventory 55	An instrument to measure HRQL in epilepsy surgery patients.	Weighted total score, three composite scores (Physical, mental and cognitive role) and eleven subscale scores:	55	Various today/last week/last year/4 weeks	Various response formats. (‘strongly agree’ to strongly disagree’, ‘often’ to ‘not at all’, yes/no)	[38,39]
			1. Emotional wellbeing				
			2. Role limitations due to emotional problems				
			3. Energy/fatigue				
			4. Social function				
			5. Pain				
			6. Physical function				
			7. Role limitations due to physical problems				
			8. Health perceptions				
			9. Cognitive function				
			10. Role limitations due to memory problems				
			11.Overall quality of life				
ICI	Ictal Consciousness Inventory	A quantitative assessment of the level of awareness and content of ictal consciousness.	Two sub-scale scores:	20	During a single seizure	Three point scale: ‘no’; ‘yes, a bit (yes, vaguely)’; ‘yes, much (yes, clearly)’.	[40]
			1. Level of consciousness (ICI-L)				
			2. Content of consciousness (ICI-C)				
IES	Impact of Epilepsy Scale	Developed to assess the impact of epilepsy and antiepileptic drug therapy on an individual’s relationship with friends and family, social life, employment, health, self-esteem, plans for the future, and standard of living.	Total impact score.	8	No recall period.	Four point scale ranging from ‘not at all’ to ‘a lot’	[41,42]
LAEP	Liverpool Adverse Events Profile	An instrument to measure total side effects burden of a medical regimen. It was developed to evaluate the most common negative side effects reported by patients taking AEDs. The AEP evaluates the interiactal state. It's widely used alone and is also part of the Liverpool Battery.	Total score.	19	Past 4 weeks	Four point scale ranging from ‘never a problem’ to ‘always or often a problem’	[43-49]
				Space to rate up to 3 additional AEs.			
LSSS	Liverpool Seizure Severity Scale 2.0 (2001)	A scale designed to quantify patient’s own perceptions of seizure severity.	Weighted most severe ictal effects score.	12	Past 4 weeks	Various scales referring to the content of each question ranging from 4 to 6 options. For example ‘I always feel sleepy’ to ‘I never feel sleepy’	[17,44,50-53]
NDDI-E	Neurological Disorders Depression Inventory for Epilepsy	An instrument to detect depression in epilepsy patients.	Total score.	6	Past two weeks	Four point frequency scale ranging from ‘always or often’ to ‘never’.	[54]
NEWQOL	Quality of Life in Newly Diagnosed Epilepsy	An instrument to measure aspects of quality of life postulated as being important for patients recently diagnosed with epilepsy.	Eight subscale scores and single item scores:	93	Varies from 'How you are now' to 'In the last year'	Various response formats including 4 and 5 point scales ranging from ‘no problem’ to ‘a serious problem’ or ‘severely restricted’ to ‘not restricted at all’	[25]
			1. anxiety				
			2. depression				
			3. social activities,				
			4. symptoms,				
			5. locus of control/mastery,				
			6. neuropsychological problems (subscales of fatigue, memory, concentration, motor skills, reading),				
			7. social stigma*, worry				
			8. work limitations.				
			Several single-item measures:				
			•general health				
			•number of seizures				
			•social limitations				
			•social support				
			•ambition				
			•limitations				
			•health transition				
			•general limitations				
	Perceived Limitations Scale	A measure of the constraints that patients with epilepsy might experience including the sense of vulnerability to the physical consequences of seizures.	Total score.	5	No recall period	Four point scale ranging from ‘strongly agree’ to ‘strongly disagree’	[55]
	Perceived Stigma Scale	A measure of the extent to which people with epilepsy feel they are victims to prejudice, including the extent to which individuals are treated differently and inability to change the views of others.	Total score.	6	No recall period	Four point scale ranging from ‘strongly agree’ to ‘strongly disagree’	[55]
PESOS	Performance, subjective evaluation and socio-demographic data	A test-battery for assessing the severity of epilepsy, epilepsy related quality of life, restrictions in daily life and psychosocial problems.	Separate scores for each instrument in the battery:	69	Various recall periods	Various response options	[56]
			1. Restrictions in daily life^				
			2. Epilepsy related fear^$^				
			3. Stigma				
			4. Emotional adaptation				
			5. Problems at work				
			6. Problems at school				
			7. Problems with parents				
PNS	Portland Neurotoxicity Scale	An instrument to measure commonly experienced adverse events associated with AEDs.	Total score and two subscale scores:	15	Past few weeks	Nine options ranging from ‘no problem’ to ‘severe problem’	[57]
			1. Cognitive toxicity				
			2. Somatomotor toxicity				
QOLIE-10	Quality of life in epilepsy 10	A brief measure of overall quality of life for patients with epilepsy.	Total score and three subscale scores:	10	Past 4 weeks	Various 5 point scales including ‘not at all‘to ‘a great deal’ and ‘not at all fearful’ to ‘extremely fearful’	[58,59]
			1. Epilepsy effects				
			2. Mental health				
			3. Role functioning				
			Alternatively scores are calculated for the 7 domains of the QOLIE-31, although for the QOLIE-10, 5 of the 7 subscales are scored from single items.				
QOLIE-31	Quality of life in epilepsy 31	Overall quality of life for patients with epilepsy.	Total weighted score and seven subscale scores:	31	Past 4 weeks	Various response formats including five and six point scales ranging from ‘very fearful’ to ‘not fearful at all’ or all the time’ to ‘none of the time’	[44,59-63]
			1. Seizure worry				
			2. Overall quality of life				
			3. Emotional well-being				
			4. Energy/fatigue				
			5. Cognitive functioning				
			6. Medication effects				
			7. Social functioning				
QOLIE-89	Quality of life in epilepsy 89	Overall quality of life for patients with epilepsy	Total weighted score and 17 subscale scores:	89	Past 4 weeks	Various response formats including yes/no or 5 point scales ranging from ‘excellent’ to ‘poor’ or ‘all the time’ to ‘none of the time’	[44,60-67]
			1. Seizure worry				
			2. medication effects				
			3. health perceptions				
			4. health discouragement				
			5. work/driving/social function				
			6. language				
			7. attention/concentration				
			8. memory				
			9. overall QOL				
			10. emotional wellbeing				
			11. role limitations: emotional				
			12. role limitations: physical				
			13. social isolation,				
			14. social support				
			15. energy/fatigue				
			16. physical functioning				
			17. pain.				
SEALS	Side Effect and Life Satisfaction Inventory	A self-report questionnaire designed to measure satisfaction with AED therapy.	Total score and five subscale scores:	38	Past week	Four point scale ranging from ‘never’ to ‘many times’.	[68-71]
			1. Cognition				
			2. Dysphoria				
			3. Tiredness				
			4. Temper				
			5. Worry				
SHE	Subjective Handicap of Epilepsy	Measures patient’s subjective handicap of epilepsy based on the WHO concept of handicap. It is recommended for use in studying the long-term consequences of medical, psychosocial and surgical interventions in epilepsy.	Six subscale scores:	32	Past 6 months, except for the change scale which is the last year	Various 5 point scales. For example, ‘Much better’ to ‘Much worse’ or ‘Very often’ to ‘never’.	[72]
			1. Work and activities	Some responses are optional based upon the patient’s situation.			
			2. Social and personal				
			3. Physical				
			4. Self-perception				
			5. Life satisfaction				
			6. Change				
SIDAED	Assessing side effects of AED treatment	An instrument to assess the duration and severity of adverse events that are possibly AED related.	Total score.	46	No recall period	Four point severity scale ranging from ‘no problem’ to ‘serious problem’ and a three point duration scale ranging from ‘since a few weeks’ to ‘half a year or longer’.	[73-77]
			Adverse events can also be grouped in 10 categories				
			1. General CNS				
			2. Behaviour (increased irritability)				
			3. Depressive symptoms				
			4. Cognitive function				
			5. Motor problems and coordination				
			6. Visual complaints				
			7. Headache				
			8. Cosmetic and dermatological complaints				
			9. Gastrointestinal complaints				
			10. Sexuality and menses				
SSQ	Seizure Severity Questionnaire	An instrument designed to assess seizure severity as a treatment response. The measure asks about events before, during and after a seizure and covers bother, severity and frequency of seizures.	Three subscale scores:	22	Past 4 weeks	Various response formats. Format is primarily seven point scales referring to frequency, ranging from ‘never’ to ‘always’, bother, ranging from ‘no bother at all’ to ‘very bothersome’ and severity ‘very mild’ to ‘very severe’, There are also yes/no responses.	[78]
			1. Seizure severity				
			2. Overall assessment				
			3. Change after alteration of treatment				
WPSI	Washington Psychosocial Seizure Inventory	Provides absolute and relative estimates of psychosocial functioning.	Eight subscale scores:	132	No recall period	Yes/no responses reflecting self-perceived feelings and actions.	[79-81]
			1 .Family background				
			2. emotional adjustment				
			3. interpersonal adjustment				
			4. financial status				
			5. adjustment to seizures				
			6. medicine				
			7. medicinal management				
			8. overall psychosocial functioning				

*Stigma of epilepsy scale has been used as a stand-alone PRO instrument to measure epilepsy patient’s perception of stigma. It comprises 3 items forming a total score [81-84].

^ Restrictions in daily life has been used as a stand-alone PRO instrument to measure social, physical and psychological dimensions that are generally stressed as central aspects of HRQL. It comprises 11 items representing three sub-scales: independent living and mobility, physical and emotional health, partnership, family and friends [56].

**$** Epilepsy Related Fears has been used as a stand-alone PRO instrument to measure fears regarding aspects of physical and social consequences. Comprising 11 items representing two sub-scales: Physical Consequences, Social Consequences [56].

AED: anti-epileptic drug.

Instruments vary in terms of length, ranging from five items (Perceived Limitations Scale) to 132 items (WPSI). Nine of the PROs do not have a defined recall period (e.g. ABNAS, SIDAED), two refer to the time of an epilepsy attack (Attack Symptom Measure, ICI). Most specify a recall period varying from *now* (EPI-QOL) to *six months* (SHE), with five PROs referring to multiple recall periods within the same PRO (e.g. EPI-QOL, NEWQOL, SHE). Response options for most of the reviewed PROs employ a Likert approach with 3–5 options, for example the Epilepsy Psycho-Social Effects Scale has a five point Likert scale ranging from ‘almost always or always’ to ‘never’ and the EPI-QOL has a five point response scale ranging from ‘very frequently’ to ‘not at all’. Less common is a numerical rating scale (NRS) approach with anchors, such as the ESES which has a 11 point scale ranging from 0 (I cannot do at all) to 10 (sure I can do).

The PROs provide different levels of information depending on the scoring approach, a minority of instruments provide only a total score based on the scoring of all items in the PRO (e.g. EFA Concerns Index, NDDI-E), whilst most provide a more detailed amount of information through provision of sub-scale scores (also referred to as domain scores), with the number of sub-scale scores ranging widely, from two (e.g. ICI, PNS) to 20 (Bonner Psychsoziale Skale fur Epilepsie (BPSE)). Most instruments provide between 5–15 subscale scores.

### PRO review against regulatory requirements

Table 3 provides the results of the PRO review against the regulatory requirements detailed in Table 1.

**Table 3:** Instrument review against regulatory requirements

**Instrument abbreviated name**	**Conceptual framework**	**Validity: content**		**Validity: construct**	**Reliability: test-retest**	**Reliability: internal consistency**	**Ability to detect change**	**Interpretation of change**
		**Patient input**	**Literature & clinician input**					
ABNAS	✘	Partial	✓	Partial	Partial	Partial	-	-
Attack Symptom Measure	✘	✘	✓	Partial	-	Partial	-	-
BPSE	✘	✘	✓	Partial	-	Partial	-	-
EFA	✘	✓	✘	Partial	✓	Partial	-	-
EPSES	✘	Partial	✓	Partial	Partial	-	-	-
EPI-QOL	✘	Partial	✓	✓	Partial	Partial	-	-
ESES	✘	Partial	Partial	Partial	Partial	✓	-	-
ESI-55	✘	Partial	✓	✓	-	Partial	✓	-
ICI	✘	Partial	✓	✓	-	✓	-	-
IES	✘	Partial	✓	✓	-	✘	-	-
LAEP	✘	Partial	✘	Partial	✓	✓	-	✓
LSSS	✘	✓	✓	Partial	Partial	Partial	Partial	-
NDDI-E	✘	✘	✓	✓	✓	✓	-	-
NEWQOL	✘	Partial	✓	Partial	Partial	Partial	-	-
Perceived Limitations Scale	✘	✘	✘	-	-	✘	-	-
Perceived Stigma Scale	✘	✘	✘	-	-	✘	-	-
PESOS	✘	✘	✓	Partial	Partial	Partial	✓	-
PNS	✘	✘	✓	Partial	✓	-	-	-
QOLIE-10	✘	Partial	✓	Partial	Partial	✘	Partial	-
QOLIE-31	✘	Partial	✓	Partial	Partial	Partial	✓	Partial
QOLIE-89	✘	Partial	✓	Partial	Partial	Partial	✓	Partial
SEALS	✘	Partial	✓	✓	Partial	Partial	-	-
SHE	✘	✓	✓	✓	✓	Partial	-	-
SIDAED	✘	✘	✘	-	-	-	-	-
SSQ	✘	Partial	✓	Partial	Partial	-	-	-
WPSI	✘	✘	✓	Partial	Partial	Partial	Partial	-

#### Conceptual framework

The authors did not find any published conceptual framework for any of the reviewed instruments.

#### Content validity

Three of the PROs involved patients in concept elicitation/item generation and in the evaluation of items through a cognitive debrief methodology or similar approach: the EFA, LSSS and SHE. Of these, the LSSS and SHE also had documented evidence that literature had been used to guide the instrument development and/or clinical experts were involved. Most of the PROs (n = 14) had partially involved patients in the development of the instrument, in most cases either concept elicitation OR cognitive debrief were undertaken, but not both methodologies. All but one of these 14 instruments (LAEP) had also reviewed literature and/or involved clinical experts in the instrument development. Of the 26 instruments only five did not involve literature and/or clinical experts in the instrument development process according to published information.

#### Construct validity

Seven instruments had full evidence of the construct validity of the PRO by providing hypotheses of the expected relationships between the PRO under evaluation and other clinical or PRO measures, and the hypotheses being supported by reported results. Most of the PROs only had partial evidence of construct validity (n = 16), in most cases there was no evidence of hypotheses of expected relationships being developed in advance of analysis. For some instruments this was compounded by limited testing e.g. only known groups validity was evaluated or very limited comparisons were made. The Perceived Limitations Scale, Perceived Stigma Scale and SIDAED did not have any available evidence of construct validity.

#### Reliability

Five PROs had sufficient evidence of test-retest reliability, and four PROs had sufficient evidence of internal consistency reliability, with two PROs providing sufficient evidence of both types of reliability: LAEP and NDDI-E. Thirteen PROs had partial evidence of test-retest reliability and 14 had partial evidence of internal consistency. Most of the PROs that had only partial evidence of these measurement properties had a mix of results for sub-scales in terms of achieving the required criteria (i.e. α ≥ 0.8 for internal consistency [85,86], r ≥ 0.7 for test-retest reliability) (e.g. PESOS, EPI-QOL) or there were no results provided for the PROs total score (e.g. Attack Symptom Measure). Eight PROs had no evidence of test-retest reliability (Attack Symptoms Measure, BPSE, ESI-55, ICI, Impact of Epilepsy scale (IES), Perceived Limitations Scale, Perceived Stigma Scale and SIDEAD) and four instruments had no evidence of internal consistency reliability (Epilepsy Psycho-Social Effects Scale, Portland Neurotoxicity Scale, SIDAED and SSQ).

Four PROs had evidence of testing for internal consistency but failed to reach the required standard of α ≥ 0.8 for all reported scales including any total score: Quality of Life in Epilepsy 10 (QOLIE 10), Perceived Stigma Scale, Perceived Limitations Scale and IES. The IES has an internal consistency of 0.65 which increases to 0.82 if one of the 8 items is removed, but later publications are based on either the eight item version or a 10-item version [42,87] for which no published psychometric validation evidence could be found. The Perceived Limitations Scale had a notably low internal consistency (α = 0.55) with the Perceived Stigma Scale getting close to the required standard (α = 0.75) [55]. The reported alpha values for the three empirically derived factors from the QOLIE-10 (epilepsy effects, mental health scale, role function) do not meet the criterion thresholds [58]. These three subscales are not the usual scores derived from the QOLIE-10. Researchers more commonly report the same seven subscales as the QOLIE-31 (five of which have only one item in the QOLIE-10) and/or a total QOLIE-10 score [59,88,89] for which no evaluation of internal consistency has been published.

#### Ability to detect change

Four PROs had full evidence of ability to detect change and reported the results of analysis undertaken with the specific aim of testing within-group responsiveness for all sub-scales as well as total score (as appropriate): ESI-55, Performance, subjective evaluation and socio-demographic data (PESOS), Quality of Life in Epilepsy 31 (QOLIE-31) and Quality of Life in Epilepsy 89 (QOLIE-89). Three PROs provided partial evidence of ability to detect change: LSSS, QOLIE-10 and WPSI; for example analysis was conducted on a previous version of the PRO (LSSS) or a non-empirically derived scale structure (QOLIE-10 results based on sub-scales taken from the QOLIE-31). For most of the PROs (n = 19) this measurement property had not been investigated.

#### Interpretation of change

The LAEP was the only PRO that had fully documented evidence of the minimally important difference (MID) of the scale. This was evaluated in a study that was designed to assess the magnitude of change in the LAEP and other PROs in order to exclude chance or error at various levels of certainty in patients with medically refractory epilepsy through application of a Reliable Change Index analytic approach [44]. Two PROs had partial evidence of MID; the MID investigation for the QOLIE-31 provided evidence for the total score but none of the sub-scales and for the QOLIE-89 results were provided for selected sub-scales and total score. No anchor-based values for interpreting change were reported for any of the reviewed instruments, and none reported responder definitions for an epilepsy population according to the FDA’s requirements around establishing responder definitions (see Table 1).

## Discussion

This review sought to evaluate epilepsy-specific PRO instruments against FDA regulatory standards and to identify gaps in the development process of the instruments that would need to be addressed prior to regulatory review. Twenty-six (26) epilepsy-specific PRO instruments were identified and reviewed.

This review identified that the SHE and NDDI-E met more of the regulatory requirements in terms of measurement properties, with both scales meeting four of the eight measurement properties evaluated. These two PROs lacked a published conceptual framework and require further evidence of ability to detect change and interpretation of change. In addition, the NDDI-E requires further evidence of patient input and the SHE requires further evidence of internal consistency reliability. Internal consistency fell below the required standard (α ≥0.8) [85] for some of the SHE’s sub-scales, further developmental work might be needed to increase the internal consistency of this scale (e.g. item removal, development of supplemental items, item re-wording, revised scale structure/conceptual framework). The SHE and NDDI-E were designed for different purposes. The SHE is a measure of patient’s subjective handicap of epilepsy based on the World Health Organisation (WHO) concept of handicap, providing six subscale scores: work and activities; social and personal; physical; self-perception; life satisfaction; and change. The lack of focus on signs and symptoms in the SHE is likely to make it unfavourable from the FDA perspective as an instrument to support a PRO label claim. The same is true of other PROs evaluated in this review. The NDDI-E is a short instrument designed to detect depression in epilepsy patients, providing a total score. Whilst the NDDI-E has been used as an outcome measure in clinical trials [90,91], this may have been inappropriate as it was designed as a screening tool, and therefore its ability to detect change is not only unknown but potentially unlikely given that it was not designed to capture change.

The LAEP, ICI and ESI-55 all met requirements for three of the eight measurement properties. The LAEP in terms of test-retest reliability, internal consistency and interpretation of change; the ICI in terms of literature and clinician input, construct validity and internal consistency; and the ESI-55 in terms of literature and clinician input, construct validity and ability to detect change. Therefore all three instruments lack a published conceptual framework and required further evidence in terms of patient input, as well as specific gaps for each PRO.

When considering PROs with at least partial evidence of measurement properties, a different group of PROs come to the forefront: the QOLIE-31 and QOLIE-89 met or partially met requirements for seven out of the eight measurement properties, and the LSSS met or partially met requirements for six of the measurement properties. However, of concern for the QOLIE-89 is that seizure free patients did not score significantly higher than the most severe group on nine of the sub-scales [64] and test-retest reliability was <0.7 for four of the sub-scales [64]. Of concern for the QOLIE-31 is that internal consistency was <0.8 for four sub-scales [60] (although at 0.77-0.79 they were very close to this threshold) and test-retest fell below 0.7 for one sub-scale [60]. Questions are raised over the LSSS because of gaps in the evidence for reliability and validity.

Of particular concern from a regulatory perspective are the Perceived Limitations Scale, Perceived Stigma Scale and the SIDAED, all of which failed to achieve even partial evidence for any of the eight measurement characteristics. Notably, the Perceived Limitations Scale and Perceived Stigma Scale failed to achieve required standards for internal consistency when tested (no evidence of testing for this for the SIDAED). No other psychometric properties were tested for these three PROs.

Content validity is defined by the FDA as ‘… the extent to which the instrument measures the concept of interest’, with evidence being supported through the conduct of qualitative studies to demonstrate that the items and domains of an instrument are appropriate and comprehensive relative to its intended measurement concept, population and use [5]. Importantly, whilst all the evidence criteria in this review are considered to be required by the FDA (with the exception of responder definitions which are recommended) the FDA make a clear statement that ‘It is important to establish content validity before other measurement properties are evaluated’. The FDA will review the process for evidence of content validity in terms of item generation, data collection method and instrument administration mode, recall period, response options, instrument format, instructions and training, patient understanding, scoring of items and domains, and respondent and administration burden. The evaluation of content validity in this review has been an assessment of process rather than an evaluation of the evidence for content validity, with access restricted to published information rather than the detailed qualitative results that would be required for FDA assessment of content validity. No published conceptual framework was identified for any of the instruments; however for nearly all of the reviewed PROs there is sufficient information regarding how items group into domains/concepts to be able to develop a conceptual framework. Compared to other deficiencies, this may not be hard to overcome. Providing empirical evidence in support of the conceptual framework, would be a greater challenge.

In terms of evidence gathered through psychometric testing, the review identified common pitfalls. In relation to testing for construct validity, the FDA require that *a priori* hypotheses are tested concerning logical relationships that should exist with measures of related concepts or scores. This was an area where most PROs failed to meet the criteria in this review. It is entirely possible that hypotheses were set in advance of analysis, but not reported in the published manuscript and it is strongly encouraged that this information is shared in publications reporting the psychometric properties of PROs. In terms of test-retest reliability and internal consistency, it is necessary to provide evidence for all domains and total scores (if applicable). Again this was a common area where PROs failed to meet the criteria in this review.

Although several PROs had evidence from clinical studies that the PRO measured change in an epilepsy population, few PROs had evidence that ability to detect change had been specifically investigated. One likely reason for this is that this measurement property is harder to evaluate in terms of study design as it means undertaking a longitudinal study of a patient group expected to improve/deteriorate. However, this is an essential measurement property, particularly when considering that the PROs are to be incorporated into clinical studies designed to test the effectiveness of a treatment for epilepsy.

Even fewer PROs had evidence for interpreting change or empirically derived responder definitions. The responder definition may vary by target population or other clinical trial design characteristics and therefore the FDA will evaluate a PROs responder definition in the context of each clinical trial. Evidence towards a PROs MID can contribute to the responder definition, and so this review sought to identify any evidence of statistically derived or anchor-based MID and/or responder definition, and found that only the LAEP was able to demonstrate empirically derived evidence of the measurement property.

One reoccurring problem found through the course of this review is that of several versions of one PRO instrument. Great care has to be taken when evaluating published evidence on the development and psychometric validation of a PRO instrument that the same version of the PRO is being referred to. By way of example, the ESES was originally developed to be a 25-item instrument, with evidence for content validity, construct validity and internal consistency [24]. However, the ESES was later updated to include an additional eight items to further assess self-efficacy associated with lifestyle issues with limited details around this update being published. In these instances, the evidence provided for earlier versions of the instrument were considered ‘partial’ evidence in Table 3 as this earlier evidence is likely to indicate how the revised instrument might perform. However, from a regulatory perspective the FDA would need to see full evidence for the developmental history and measurement properties of the revised instrument, assuming it is the revised version that is currently available for use in clinical trials.

It is vital to consider these findings in the broader context of PRO label claims in the US. The review of the current state of PRO label claims granted for new molecular entities approved 2006–2010 [18] found that of 116 products identified, 24% were granted PRO claims of which 86% were for symptoms and of these 38% were pain related. The proportion of new molecular entities and biologic license applications with PRO label claims has decreased slightly from 30% 1997–2002 to 24% 2006–2010. PRO label claims for non-primary endpoints were uncommon, with occurrence of symptoms the mostly commonly reported PRO label claim granted. The majority of accepted claims were supported by simple scales such as visual analogue scale (VAS), numeric rating scale (NRS), or symptom diaries, or on the basis of measures that have been traditionally accepted by the reviewing divisions. Within this context none of the reviewed epilepsy instruments are ‘simple scales’ i.e. VAS, NRS or symptom diaries. The reviewed PRO instruments do not appear to be measures that are ‘traditionally accepted by the reviewing divisions’ as none were identified in labels for PRO epilepsy treatments approved 2006–2010. The level of evidence required to support a desired label claim on the basis of the reviewed PRO instruments is of substantial importance as the reviewed instruments are not the typical PRO instruments being seen to support NME and BLA product approvals in the US.

It is worth considering the extent to which the evaluated PROs would be suitable to support regulatory approval of medicines in Europe through a regulatory review conducted by the EMA. The EMA has been less prescriptive in terms of their requirements for PROs, with one reflection paper published in 2005 for HRQL instruments, but nothing extended more generally to PROs. In the absence of clear guidelines from the EMA, it is difficult to determine which of the evaluated PROs would be well received by the EMA. However, whilst both the FDA and EMA require PROs supporting regulatory approvals to be validated and reliable, the EMA is more likely to accept and encourage the use of well-known, commonly used PROs than the FDA. The EMA also places less emphasis on qualitative evidence of content validity. Therefore PROs that are likely to be well received by the EMA will need to have demonstrated evidence of the psychometric properties of the PROs, particularly where this evidence is published in peer-review publication.

A limitation to this research is that the review was conducted on published information. It is frequently the case that documentation on the development process for PRO instruments is not published, particularly for older PRO instruments which were developed at a time where there were less publication options for PRO development manuscripts. It is likely that there are more details on the development of the reviewed instruments that have not been considered in this review. An important step for anyone considering the use of the PRO to support a label claim is to contact the instrument developer to see if further information can be made available to address seeming gaps in the evidence, which if available will reduce the need to conduct further research to gather evidence in support of the PRO instrument.

## Conclusions

This systematic review of 26 epilepsy-specific PRO instruments, evaluated to the standards set out in the FDA guidance [5] indicates that none of the identified instruments have the full evidence required by the FDA to support the label claim, and all require further research to support their use as an endpoint. This may at least partially explain the lack of PRO label claims in support of epilepsy products. The SHE and NDDI-E have the fewest gaps that would need to be addressed through additional research prior to any FDA regulatory submission, although the NDDI-E was designed as a screening tool and is therefore unlikely to be suitable as an instrument for capturing change in a clinical trial and the SHE lacks the conceptual focus on signs and symptoms favoured by the FDA.

## Abbreviations

AEDs: Antiepileptic drugs; ABNAS: Aldenkamp-Baker neuroassessment Schedule; BPSE: Bonner psychsoziale skalen fur epilepsie; EFA: Epilepsy foundation of America; EMA: European Medicines Agency; EPSES: Epilepsy psycho-social effects scale; ESES: Epilepsy self-efficacy scale; ESI-55: Epilepsy surgery inventory 55; FDA: Food and Drug Administration; HRQL: Health related quality of life; ICI: Ictal consciousness inventory; IES: Impact of epilepsy scale; LAEP: Liverpool adverse events profile; LSSS: Liverpool seizure severity scale; MID: Minimally important difference; NDDI-E: The neurological disorders depression inventory for epilepsy; NEWQOL: Quality of life in newly diagnosed epilepsy; NRS: Numerical rating scale; PESOS: Performance, subjective evaluation and socio-demographic data; PNS: Portland neurotoxicity scale; PRO: Patient Reported Outcome; QOL: Quality of life; QOLIE: Quality of life in epilepsy; QOLIE-10: Quality of life in epilepsy 10; QOLIE-31: Quality of life in epilepsy 31; QOLIE-89: Quality of life in epilepsy 89; SEALS: Side effect and life satisfaction; SHE: Subjective handicap of epilepsy; SIDAED: Assessing side effects of AED treatment; SSQ: Seizure severity questionnaire; WHO: World Health Organisation; WPSI: Washington psychosocial seizure inventory

## Competing interests

The authors are employees of Oxford Outcomes, an ICON Plc. company. Oxford Outcomes received payment from Eisai for conducting the review and writing the manuscript. Eisai paid the article-processing charge.

## Authors’ contributions

AN: Study design, contributed to designing the search strategy, reviewed abstracts, developed the review criteria, reviewed the PRO instruments according to the criteria, led on manuscript development. CK: Study design, designed the search strategy, reviewed abstracts, developed the review criteria, reviewed the PRO instruments according to the criteria, contributed to manuscript development. KB: Contributed to designing the search strategy, reviewed abstracts, developed the review criteria, reviewed the PRO instruments according to the criteria, assisted with manuscript development. DW: Reviewed PRO instruments, reviewed the manuscript content. All authors read and approved the final manuscript.

## Supplementary Material

Additional file 1 Listing of search terms used through Embase and Medline. Click here for file
