# Supplementary material for: Patient Reported Outcome (PRO) assessment in epilepsy: a review of epilepsy-specific PROs according to the Food and Drug Administration (FDA) regulatory requirements
Source: Health Qual Life Outcomes. 2013 Mar 11;11:38. doi: 10.1186/1477-7525-11-38 (PMC3606363; doi:10.1186/1477-7525-11-38)
Supplement: Additional file 1 — Listing of search terms used through Embase and Medline. [file 1477-7525-11-38-S1.pdf]

Additional file 1

Medline search terms

1	epilepsy/
2	seizure/
3	1 or 2
4	patient reported outcome$.mp.
5	patient outcome report.mp.
6	questionnaire$/
7	instrument$.mp.
8	"self assessment (Psychology)"/
9	self report/
10	instrument validation.mp.
11	psychometrics/
12	health outcome measure$.mp.
13	health outcome questionnaire$.mp.
14	health status indicator.mp.
15	inventory/
16	measure/
17	or/4-16
18	3 and 17
19	limit 18 to (english language and humans and yr="2000 - Current")

Embase search terms

1	epilepsy/
2	seizure/
3	1 or 2
4	patient reported outcome$.mp.
5	patient outcome report.mp.
6	health outcome measure$.mp.
7	health outcome questionnaire$.mp.
8	self report/
9	measurement/
10	questionnaire/
11	instrument/
12	instrument validation/
13	self assessment.mp.
14	inventory/
15	health status indicator.mp.
16	psychometry/
17	or/4-16
18	3 and 17
19	limit 18 to (human and english language and yr="2000 - Current")
